# Identification of common horsetail (*Equisetum arvense* L.; Equisetaceae) using Thin Layer Chromatography versus DNA barcoding

**DOI:** 10.1038/srep11942

**Published:** 2015-07-13

**Authors:** C. Haris Saslis-Lagoudakis, Sam Bruun-Lund, Natalie E. Iwanycki, Ole Seberg, Gitte Petersen, Anna K. Jäger, Nina Rønsted

**Affiliations:** 1 Evolutionary Genomics Section, Natural History Museum of Denmark, Sølvgade 83S, Copenhagen, DK-1307, Denmark; 2 Natural Products Research, Department of Drug Design and Pharmacology, University of Copenhagen, Universitetsparken 2, Copenhagen, DK-2100, Denmark

## Abstract

The global herbal products market has grown in recent years, making regulation of these products paramount for public healthcare. For instance, the common horsetail (*Equisetum arvense* L.) is used in numerous herbal products, but it can be adulterated with closely related species, especially *E. palustre* L. that can produce toxic alkaloids. As morphology-based identification is often difficult or impossible, the identification of processed material can be aided by molecular techniques. In this study, we explore two molecular identification techniques as methods of testing the purity of these products: a Thin Layer Chromatography approach (TLC-test) included in the European Pharmacopoeia and a DNA barcoding approach, used in recent years to identify material in herbal products. We test the potential of these methods for distinguishing and identifying these species using material from herbarium collections and commercial herbal products. We find that both methods can discriminate between the two species and positively identify *E. arvense*. The TLC-test is more cost- and time-efficient, but DNA barcoding is more powerful in determining the identity of adulterant species. Our study shows that, although DNA barcoding presents certain advantages, other established laboratory methods can perform as well or even better in confirming species’ identity in herbal products.

Tens of thousands of plant species are used medicinally^1^ and a substantial portion of the world’s population depends on traditional medicine^2^. In recent decades, public interest in herbal products has grown^3^,^4^,^5^, but these products are not always regulated. The safety of herbal products can be compromised through accidental adulteration, misidentification and deliberate contamination^6^,^7^, which can lead to severe side effects due to the presence of toxic compounds^8^. This creates a need for authentication of species included in these products. The qualitative and quantitative composition of herbal products is regulated by international and national monographs such as the European Pharmacopoeia^9^, which presents a series of monographs for herbal products, including recommended tests for identification and quality. These tests are often based on morphology. However, macroscopic or microscopic identification of plant species requires considerable expertise to differentiate between closely related or similar looking species. Furthermore, morphological characters may be indistinguishable in bulk, pulverised or otherwise processed material^10^,^11^.

To circumvent these problems, most monographs define a maximum allowance of foreign matter often based on a Thin Layer Chromatographic (TLC) test using chemical markers allowing distinction between the correct species and other, potentially toxic species^12^,^13^. However, such chemical markers or fingerprinting analyses have certain drawbacks. First, it is often difficult to find chemical markers that are unique to the target species. Different species can produce the same marker, hindering species’ identification. Second, chemical composition can demonstrate considerable intraspecific variability depending on season, growth, storage conditions and harvesting process^14^. Third, herbal products are sometimes spiked with synthesised compounds^15^. In these cases, the TLC-test may lead to false species’ identification.

An alternative method that has been used to identify components of herbal products is DNA barcoding^10^,^11^,^16^,^17^,^18^,^19^. DNA barcoding relies on sequencing of short fragments of the genome, which are unique to the target species^20^. The DNA sequences from the product are compared to a reference database, based on which the identity of the species can be confirmed^21^,^22^. DNA-based identification methods have often revealed adulteration in traditional medicinal preparations and herbal products. For example, potentially toxic *Ephedra* L. and *Asarum* L. material was found in Traditional Chinese Medicinal products administered in Australia^23^, and several adulterant plant species were found in herbal products from North America^17^. Nevertheless, DNA barcoding also has limitations. First, depending on the condition of the plant material, amplification of the target DNA marker may not be possible. Second, DNA barcodes might show low interspecific variability, particularly among closely related species. Finally, because DNA barcoding relies on the presence of a reference database, the absence of a species from the database will impede its identification success^19^. Despite its limitations, DNA barcoding has often been discussed as the primary method of molecular identification of plants in the last decade^11^,^16^,^22^.

In this study, we explore molecular identification of the genus *Equisetum* L. (Equisetaceae), also known as horsetails. The genus comprises 15 species and has a more or less cosmopolitan distribution^24^,^25^. *Equisetum arvense* L. is used traditionally against numerous conditions^26^ and many *E. arvense* herbal products are sold on the market mainly against urinary and renal conditions^27^, as well as skin, hair and nail remedies, potentially due to the species’ high silica content^28^. The separation of *E. arvense* from other *Equisetum* species – especially *E. palustre* L. that contains toxic levels of the pyridine alkaloid palustrine – is challenging^29^,^30^, particularly based on microscopic examination of commercial herbal products. Therefore, the European Pharmacopoeia monograph for the common or field horsetail, *E. arvense,* includes a TLC-test (Identification C) that tests for its positive identification, including a test for foreign matter from *E. arvense*. However, it is not clear whether this test can positively identify either *E. arvense* or *E. palustre* among other morphologically similar *Equisetum* species, several of which overlap geographically with *E. arvense*^31^. This is a potential problem because palustrine is not specific to *E. palustre*, but it is found in other horsetail species. An early study detected palustrine in *E. arvense* and *E. hyemale* L.^32^. A later study did not detect it in *E. arvense*, *E. telmateia* Ehrh., and *E. sylvaticum* L.^33^, but a more recent compendium of poisonous plants cites palustrine and palustridine alkaloid content for *E. fluviatile* L., *E. hyemale*, *E. palustre*, *E. sylvaticum*, and *E. telmateia*^34^.

The main objective of this study was to investigate the resolution power of the European Pharmacopoeia’s TLC-test and of the DNA barcoding approach for i) distinguishing between *E. arvense* and *E. palustre* and ii) positively identifying these two species and discriminating them from other *Equisetum* species. In order to perform these investigations, we needed to have a reliable species’ delimitation. Therefore, we also reconstructed a molecular phylogeny of *Equisetum* to test currently accepted species boundaries. Our study is based on herbarium collections of wild origin, as well as exemplar herbal products from the market.

## Results

### Phylogeny of *Equisetum*

**Figure 1:**
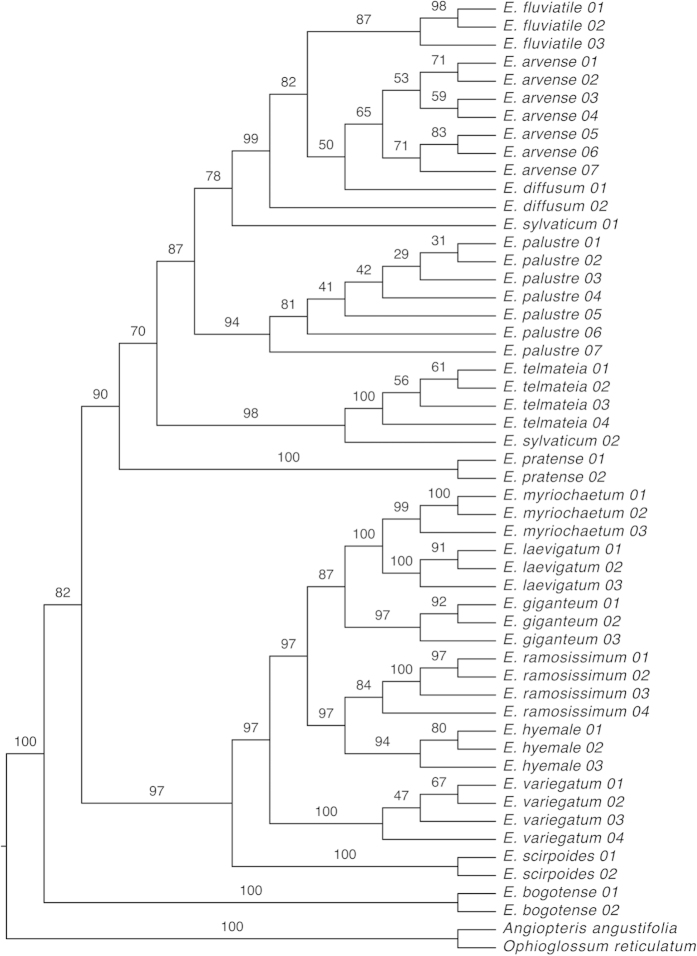
Phylogeny of *Equisetum* reconstructed with a Maximum Likelihood analysis based on five DNA markers (ITS2*, matK, rbcL, rps4, trnH-psbA*). Bootstrap support values are given above respective branches.

We reconstructed a phylogenetic tree of *Equisetum* (Fig. 1) in order to test the monophyly of the species. Previous studies have provided phylogenetic hypotheses for the genus using plastid DNA markers^35^,^36^,^37^, but these studies only included one specimen per species. The topology obtained here from nuclear and plastid markers, and including several accessions per species, largely corresponds to the topology found previously^35^,^36^,^37^. *Equisetum bogotense* Kunth is recovered as sister to the rest of the genus and not as a member of subg. *Hippochaete* (Milde) Baker. The remainder of the genus is resolved into two major clades, each comprising seven species and corresponding to the two subgenera *Equisetum* and *Hippochaete* (Fig. 1). With the exception of *E. diffusum* D. Don and *E. sylvaticum*, all species were recovered as monophyletic, including *E. arvense* and *E. palustre*. These two species are resolved in the same clade (subg. *Equisetum*), but not as sister species (Fig. 1).

### Distinction between *E. arvense* and *E. palustre*

#### Chemistry

**Figure 2:**
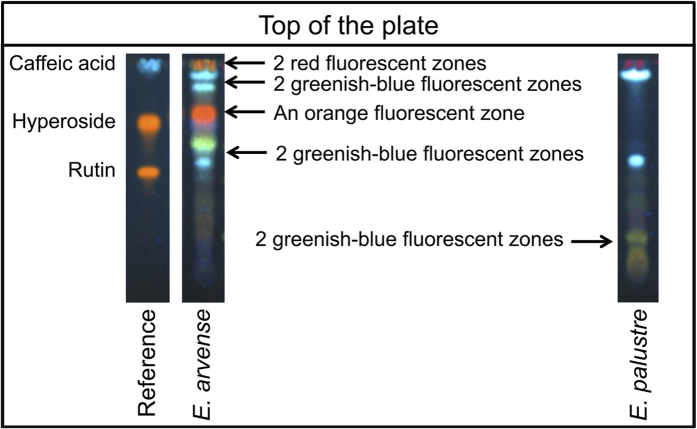
Exemplar chromatograms of *E. arvense* and *E. palustre* pointing out the combination of characters used in the European Pharmacopoeia to identify the species (four for *E. arvense* and one for *E. palustre*). Although some markers are not unique to *E. arvense*, the combination of all four traits serves for its positive identification. The reference solution is also presented.

**Figure 3:**
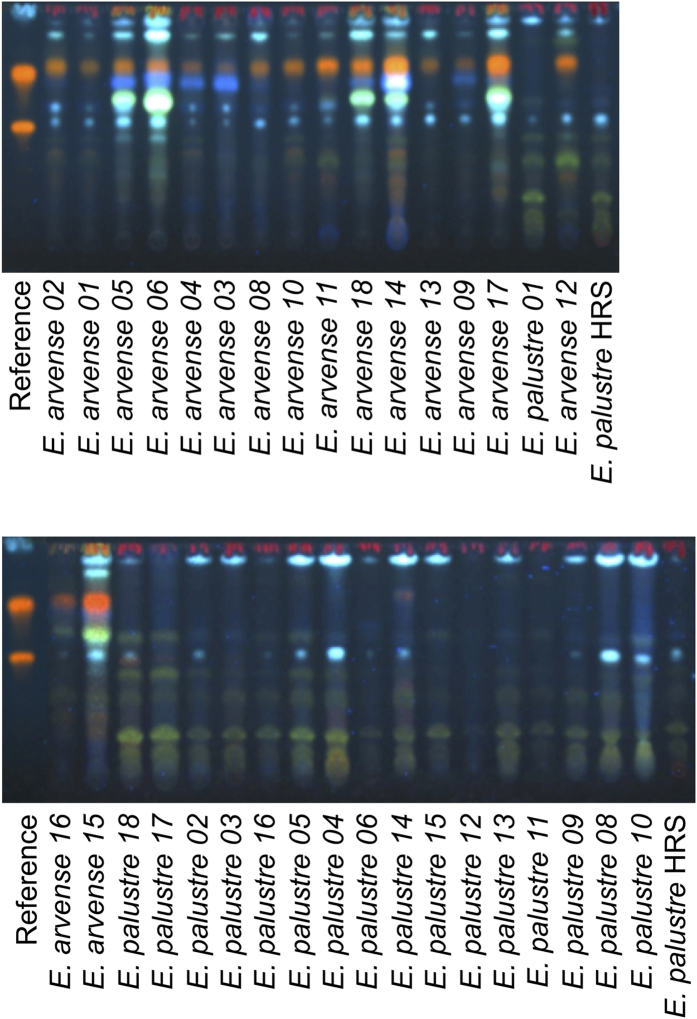
TLC chromatogram of *Equisetum arvense* and *E. palustre* accessions from natural history collections.

**Figure 4:**
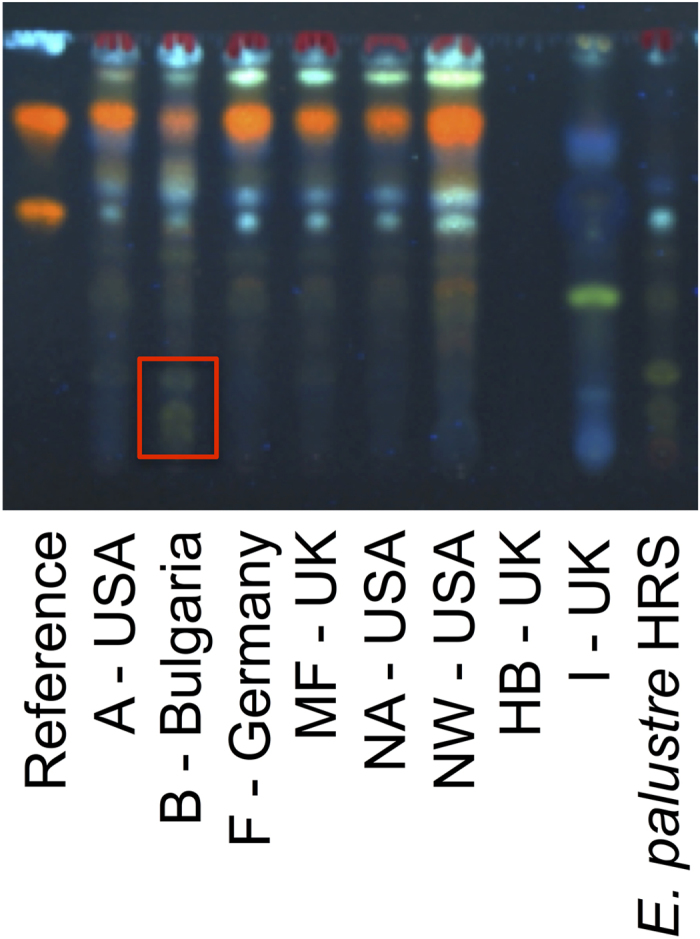
TLC chromatogram of commercial products sold as *Equisetum arvense*. We only provide the acronym of each product and its country of manufacture. The distinctive greenish-blue band area that indicates presence of *E. palustre* material is highlighted inside the red rectangle.

The distinction between the two species based on the TLC-test of the European Pharmacopoeia is based on the presence of a combination of marker bands in each species, shown in Fig. 2. The results of the TLC-test recommended by the European Pharmacopoeia are shown in Fig. 3 for the *E. arvense - E. palustre* comparison. The two bands at the bottom of the plate that are used for the identification of *E. palustre* are present in all accessions of this species, but not in any of the *E. arvense* accessions. Although some of the marker bands used to identify *E. arvense* can be found in *E. palustre* accessions, the combination of the four marker bands (Fig. 2) is not seen in any *E. palustre* accessions (Fig. 3). Therefore, the marker zones used as the distinguishing characters between the two species in the monograph (Fig. 2) could consistently distinguish between *E. arvense* and *E. palustre*. We observed a typical *E. arvense* TLC chromatogram for five out of eight commercial products included in the analysis (Fig. 4). In one product (B – Bulgaria), we observed the marker bands that are used to identify both *E. arvense* and *E. palustre* in the TLC-test of the European Pharmacopoeia, suggesting this product includes a mixture of the two species (Fig. 4). One product (I – UK) seemed to not contain any *Equisetum* material at all and another one (HB – UK) returned no chromatogram (Fig. 4).

#### DNA barcoding

**Figure 5:**
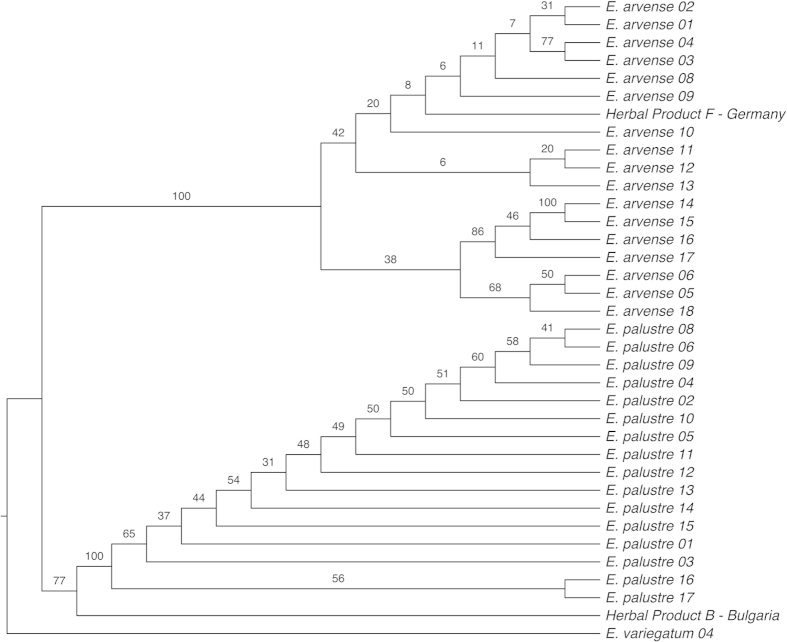
DNA barcoding of *Equisetum arvense* and *E. palustre* based on two markers (*matK* & *trnH-psbA*). The phylogenetic tree was reconstructed with a Maximum Likelihood analysis based, using *E. variegatum* as an outgroup. Bootstrap support values are given above respective branches.

The two plastid markers we used for the DNA barcoding of *E. arvense* and *E. palustre* resolve the samples into two well supported, monophyletic clades, shown in Fig. 5. We were able to amplify DNA from two of the herbal products, only; one was resolved within the *E*. *arvense* clade (BP 100). The other, which was shown to be a mixture from the TLC-test (B – Bulgaria), is recovered with the *E. palustre* (BP 77) clade (Fig. 5). For both barcoding regions, we found 36 substitutions (25 for *matK* and 11 for *trnH-psbA*) that can distinguish *E. arvense* and *E. palustre*. Some of them are unique to each species and others are shared with other species, but not between *E. arvense* and *E. palustre* (Table 1).

### Positive identification of *E. arvense* and *E. palustre*

#### Chemistry

**Figure 6:**
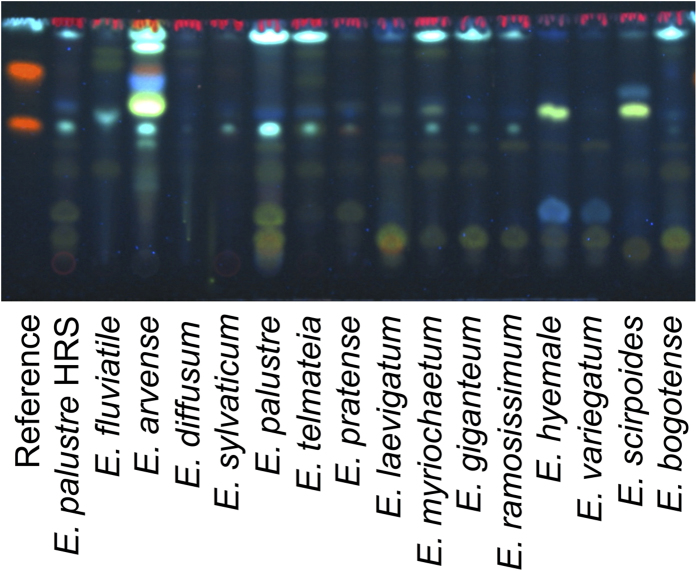
TLC chromatogram of exemplar accessions of all *Equisetum* species.

We analysed one exemplar specimen of all *Equisetum* species using the TLC-test recommended by the European Pharmacopoeia (Fig. 6). For *E. diffusum* and *E. sylvaticum*, which were not monophyletic in the DNA analysis, we could test only one sample, as the other sample did not come from our study. The TLC-test (Identification C) of the European Pharmacopoeia can positively identify *E. arvense*. Although some of the marker bands outlined in the TLC identification test for *E. arvense* (Fig. 2) are seen in the chromatograms of other *Equisetum* species, *E. arvense* is the only species with the combination of all these markers bands (Fig. 6). As shown in Fig. 6, one or two of the greenish-blue fluorescent zones used in the TLC-test to detect *E. palustre* were not detected in any other species within subg. *Equisetum*, but were present in all species in subg. *Hippochaete*. Therefore, the TLC-test of the European Pharmacopoeia cannot be used to identify *E. palustre*, because the trait of this species (Fig. 2) is shared with other *Equisetum* species as well (Fig. 6).

#### DNA barcoding

We investigated whether the two DNA markers we used as barcodes can not only differentiate *E. arvense* from *E. palustre*, but also include a combination of unique traits for these species, which can be used to successfully identify them from all other *Equisetum* species. Table 1 shows that there are unique substitutions in these two markers, the combination of which can positively identify both *E. arvense* and *E. palustre* from other *Equisetum* species. For *matK*, we found no substitutions to be unique for *E. arvense*, but five substitutions were unique for *E. palustre* (Table 1). For *trnH-psbA*, one substitution was unique for *E. arvense* and two for *E. palustre* (Table 1). Regarding the identification of material in herbal products, DNA sequences from one product (F – Germany) show the combination of characters that can identify *E. arvense*. For the other product (B – Bulgaria), we only managed to amplify *matK*. This sequence is actually a chimeric sequence (several doubles peaks are observed in the DNA chromatograms), showing some characters that are characteristic of *E. arvense* and some of *E. palustre*.

## Discussion

Some *Equisetum* species are morphologically quite variable and can be difficult to identify based on morphology alone^29^,^30^. To the untrained eye, *E. arvense* may superficially resemble other species within subgenus *Equisetum*, including *E. palustre*, as well as *E. pratense*, *E. fluviatile*, *E. telmateia* and *E. diffusum*. Positive identification of material lacking strobili, or where information about dimorphism is lacking, may be challenging even for trained botanists, as micro-morphological or anatomical characteristics may be required to separate some species, e.g. *E. arvense* and *E. palustre*^38^. Within their respective ranges, taxa sharing similar morphological characters, such as *E. arvense* and *E. palustre*, may be found co-occurring in the same habitat^31^. A further complication to field-identification is that *E. arvense* is known to form hybrids with *E. palustre* (*E*. × *rothmaleri* C.N. Page) and *E*. *fluviatile* (*E* × *littorale* Rupr.)^39^,^40^, with morphological and chemical traits that are intermediate between the parent taxa^30^,^40^.

Due to the risk of misidentification or adulteration of *E. arvense* with *E. palustre*, laboratory techniques are needed for the quality control of herbal products of *E. arvense*. The European Pharmacopoeia has devised a simple method using TLC (Identification C) to distinguish the two species^9^, and we found this test to be straightforward and consistent. It can confirm that the material is from *E. arvense*, through a combination of marker bands unique to this species (Figs 2,3 and 6). Further, as shown in Fig. 3, the two greenish-blue bands at the bottom are present in all *E. palustre* accessions, but none of the *E. arvense* accessions. The presence of these bands can be used as an indication of adulteration with *E. palustre*, but the identity of the adulterant is not confident, because these bands are also found in other *Equisetum* species besides *E. palustre* (Fig. 6). Also, even in the case of absence of these bands, a partial adulteration with another *Equisetum* species that does not demonstrate them in the chromatogram (Fig. 6) cannot be ruled out.

The current TLC-test is testing for the presence of kaempferol glucosides (flavonoids), instead of directly testing for the presence of alkaloids. We tested for alkaloids using the material which had already been extracted for the flavonoid analysis. This method could only detect alkaloid bands (two bands) present in the reference *E. palustre* HRS and the *E. palustre* accession used on the TLC-test across the genus, whereas possible alkaloids present in other *Equisetum* species were below the detection limit of this method (results not shown). We suggest that a method testing directly for alkaloids be developed and included in the monograph.

DNA barcoding may be an alternative or supplementary method to identify material in herbal products with higher certainty^17^,^18^,^19^,^41^. We found that two plastid markers can successfully distinguish between *E. arvense* and *E. palustre*. In total, there are 36 characters (25 for *matK* and 11 for *trnH-psbA*) differentiating the two species (Table 1), and the phylogenetic analysis of these two DNA barcoding markers assigns material from these two species to two well-supported clades (Fig. 5). Further, this approach can positively identify the two species, as we found six substitutions (five for *matK* and one for *trnH-psbA*) that are unique to *E. palustre* (Table 1), allowing high confidence in the identification of this species. For *E. arvense*, we only found one unique substitution in *trnH-psbA* and none in *matK* (Table 1), making assignment of material to this species less robust. However, a number of other substitutions are only shared by *E. arvense* and its two closest relatives, *E. fluvatile* and *E. diffusum* (Fig. 1), which co-occur in Asia. Including more DNA barcoding regions that have been proposed by the Consortium for the Barcode of Life Plant Working Group^22^ could provide further discriminatory power for *E. arvense*. However, we found *rbcL* to show too little interspecific variation, and ITS2, which has been proposed for the DNA barcoding of medicinal plants^16^, did not amplify consistently in *Equisetum.* Other DNA barcoding markers that have been shown recently to perform better than the ones we used here [e.g., *ycf1*^42^] could provide more species-specific substitutions in future investigations.

We included eight commercial products claiming to be *E. arvense*, seven of which produced TLC chromatograms allowing assignment of the herbal product to either *E. arvense* or *E. palustre* following the European Pharmacopoeia’s TLC-test for foreign matter. Of these seven products, five were assigned to *E. arvense* (Fig. 4). We were only able to gather DNA sequence data for one of these samples (herbal product F - Germany), and it was confirmed to be *E. arvense* (Fig. 5). For one product (herbal product B - Bulgaria), the TLC-test showed the presence of *E. arvense* and potentially *E. palustre* material (Fig. 4). The DNA sequence data confirmed that this product is most likely a mixture, as the resulting sequence was chimeric. Although this product is resolved within the *E. palustre* clade (Fig. 5), the sequence we amplified shows a combination of substitutions characteristic of *E. arvense* and *E. palustre*. It could be adulterated, misidentified or even be of hybrid origin. The product is a tea from south-eastern Europe, an area that is a major source of commercial *E. arvense* products^27^, and where the two species co-occur, raising concerns about the risk of contamination with *E. palustre* in commercially available material presumed to be *E. arvense*. Finally, one sample (herbal product I - UK) produced a chromatogram that was different from those characteristic of any *Equisetum* species (Figs 4 and 6), suggesting the botanical material in that sample might not be *Equisetum*. Unfortunately, no DNA sequence data could be gathered from that sample.

Our objective was to explore and compare the power of the European Pharmacopoeia’s TLC-test and of the DNA barcoding approach for distinguishing between *E. arvense* and *E. palustre*, as well as for positively identifying the two species. We found both methods to be useful, however with different advantages and shortcomings. In terms of success rate of data collection, the TLC-test approach is more efficient. First and foremost, the laboratory work is less laborious and cheaper than DNA barcoding. Second, the TLC-test had a greater success rate with commercial herbal products: we obtained chromatograms for seven out of eight of these products, while the amplification success of the barcoding regions from these products was limited (only two samples). On the other hand, in terms of resolution and confidence in identification, the DNA barcoding approach is better. Although both methods can successfully discriminate between *E. arvense* and *E. palustre* and positively identify *E. arvense*, only DNA barcoding provides a combination of traits that is unique to *E. palustre* among horsetail species. However, the amplification of these barcoding markers might prove difficult in processed commercial products. Additionally, an advantage of the TLC-test is that contamination can be quantified based on the level of visibility of the greenish-blue bands on chromatograms, an aspect in which the DNA barcoding approach lacks.

Which method would we recommend as being the best? Given the pros and cons of each method, we believe that it depends on the application. Our results show that, when it comes to confirming whether an herbal product contains *E. arvense,* the TLC-test is the most cost- and time-efficient option. However, the presence of the marker bands described in the TLC-test as characteristic of *E. palustre* can be seen in cases of adulteration with other *Equisetum* species, as these bands are common within the genus (Fig. 6). Similarly, the absence of these marker bands does not guarantee that the product has not been adulterated with other *Equisetum* species, which do not show those bands (Fig. 6). Given that there is uncertainty about which *Equisetum* species produce toxic alkaloids, this could be an important shortcoming of the TLC-test. In these cases, DNA barcoding can be used as a complementary test for quality control, when possible.

Our study also highlights the immense potential of herbarium collections for a wide range of modern approaches to biodiversity research^43^,^44^, and DNA barcoding in particular^45^,^46^. The majority of the material used in this study was obtained from the collections in herbarium of the Natural History Museum of Denmark (C). The age of the material ranged from 1900–2013. We did not detect any apparent age-related difference in the intensity of the TLC chromatograms or the amplification success of the DNA markers, showing that the chemical profiles and the DNA are not substantially degraded in carefully stored collections^47^. Our findings demonstrate how available collections can be used to set up a modern framework of chemical and molecular identification of economically important species. Without conducting substantial fieldwork, we managed to sample across all *Equisetum* species, as well as within *E. arvense* and *E. palustre*, covering their geographic ranges, hence ensuring that both inter- and intra-specific variation is covered. An incidental advantage of using herbarium material is that the link between the chemical and molecular data and the voucher is established by default. Missing vouchers is a serious problem in many studies^41^ which makes replication by future researchers almost impossible^48^.

## Conclusions

Given the recent growth of the herbal products market^3^,^4^,^5^, efficient methods for regulating these products against accidental adulteration, deliberate contamination and misidentification are more relevant than ever for public healthcare^41^. We tested the European Pharmacopoeia’s *E. arvense* TLC-test for foreign matter, particularly from the closely related *E. palustre*. We also tested a DNA barcoding approach to distinguish and identify these species. We found that each method has advantages and disadvantages, but the TLC-test is the most efficient way of confirming that material in herbal products is indeed *E. arvense*. On the other hand, the DNA barcoding can be used as a complementary test to determine the identity of adulterant species, particularly *E. palustre*.

Future work can focus on systematically studying which *Equisetum* species produce toxic alkaloids, which will assist the quality control of *E. arvense* herbal products. Further, a chemical method that directly tests for the presence of alkaloids in herbal products can circumvent problems in species identification, directly testing for the quality and appropriateness for human consumption of herbal products. Additionally, the steadily dropping price of next generation sequencing techniques – which massively amplify short DNA fragments – may considerably enhance the success rates of DNA barcoding in degraded or processed material. Finally, given the presence of several putative hybrids between *E. arvense* and other *Equisetum* species, further techniques can be applied to investigate the presence of hybrid material in herbal products.

## Methods

### Plant material

For the phylogenetic reconstruction, we sampled at least one accession of each *Equisetum* species, mostly from material deposited in the herbarium of the Natural History Museum of Denmark (C), in order to produce a well-sampled phylogenetic hypothesis for the genus. From these specimens, we chose one per species for the TLC-test across *Equisetum* species. For the DNA barcoding and TLC-test of *E. arvense* and *E. palustre*, we sampled several accessions of each of the two species covering their distribution ranges to the extent possible. Additionally, we sampled eight herbal products sold on the market as *E. arvense*. Details of plant materials are listed in Supplementary Tables 1 and 2.

### DNA sequencing

Complete genomic DNA was extracted using the DNeasy Mini Plant Kit (Qiagen Ltd, Crawley, UK), following the manufacturers protocol. For the DNA barcoding of *E. arvense* and *E. palustre*, we sequenced the *trnH-psbA* spacer and the barcoding fragment of *matK*, which have been used in previous DNA barcoding studies^21^,^22^,^49^,^50^. For the genus wide analysis, we sequenced the plastid regions *rps4*, *rbcL*, the barcoding fragment of *matK*, the *trnH-psbA* spacer, and the nuclear ribosomal *ITS2* region. The *rps4* marker was amplified using primers rps5 (5^′^-ATG TCC CGT TAT CGA GGA CC T-3) and trnS (5^′^-TAC CGA GGG TTC GAA TC-3)^51^,^52^ and the *rbcL* marker was amplified with primers rbcL26F (5′-ATG TCA CCA CAA ACA GAA ACT AAA GCA AGT-3′) and rbcL1379R (5′-TCA CAA GCA GCA GCT AGT TCA GAA CTC-3′)^53^. For both these markers, we used the following PCR programme: 3 minutes of initial denaturation at 94 °C, followed by 30 cycles of 45 seconds at 94 °C, 45 seconds at 53 °C, 90 seconds at 72 °C, and a final extension for 10 minutes at 72 °C. For the *trnH-psbA* spacer region a PCR was performed using primers trnHf (5′-CGC GCA TGG TGG ATT CAC AAT CC-3′) and psbA3f (5′-GTT ATG CAT GAA CGT AAT GCT C-3′)^54^,^55^ using the following conditions: 4 minutes at 95 **°**C, followed by 48 cycles of 30 seconds at 94 °C, 40 seconds at 45 °C, 40 seconds at 72 °C, and a final extension for 5 minutes at 72 °C. For *matK*, *Equisetum* specific primers were used: matK Equisetum F (5′-ATA CCC CAT TTT ATT CAT CC-3′) and matK Equisetum R (5′-GTA CTT TTA TGT TTA CGA GC-3′) [http://www.kew.org/barcoding/update.html] with the following conditions: 4 minutes at 94 **°**C following 32 cycles of 1 minute at 94 °C, 1 minute at 46 °C, 2:30 minutes at 72 °C and a final extension for 7 minutes at 72 °C. Part of the internal transcribed spacer region (ITS2) was amplified using primers ITS3 (5′-GCA TCG ATG AAG AAC GCA GC-3′) and ITS4 (5′-TCC TCC GCT TAT TGA TAT GC-3′) from White *et al.*^56^. With the following conditions: 4 minutes at 94 **°**C following 35 cycles of 1 minute at 94 °C, 1 minute at 48 °C, 1 minute at 72 °C and a final extension for 2 minutes at 72 °C.

Reactions of 25 uL were carried out using standard procedures with 1 or 2 uL DNA template. Moreover, for *matK* and ITS DMSO was added to reduce the effects of secondary structure on primer biding. BSA was added to all reactions to enhance polymerase activity. The PCR products were purified using the Qiagen PCR purification kit (Qiagen Inc.) according to the manufacturer’s instructions. Direct sequencing of purified PCR products was either performed using BIGDYE v1.1 (Applied Biosystems, Wellesley, Massachusetts, U.S.A.) and purified sequencing products were run on an AB3130 × 1 automated sequencer (Applied Biosystems) or sent to GATC-biotech in Germany (http://www.gatc-biotech.com). Forward and reverse sequences were edited and assembled in Geneious v. 7.1.7 (http://www.biomatters.com). Alignments were conducted using the MAFFT v.7 plugin^57^ in Geneious with default options and inspected manually afterwards. Regions that were ambiguously aligned were excluded from the analyses. Genbank accession numbers for all sequences used in the study are shown in Supplementary Table 3

### Phylogenetic methods

Two matrices were assembled: (1) a genus wide matrix combining our datasets with DNA sequences from previous phylogenetic studies of *Equisetum*^35^,^36^,^37^ to achieve a sampling scheme of multiple accessions per taxon allowing test of species monophyly, and (2) an *Equisetum arvense-E. palustre* dataset for the development of the DNA barcoding methodology. All sequences were aligned with MAFFT^57^ and sequence data were analysed under the Maximum Likelihood (ML) criterion, with RAxML^58^ using the partitioned model option (five partitions – one per DNA marker) with the GTR+I+G model and running 100 bootstrap replicates^59^. *Angiopteris angustifolia* and *Ophioglossum reticulatum* were used as outgroup for the phylogenetic analysis of the genus, and *E. variegatum* was used as outgroup for the phylogenetic analysis of DNA barcodes.

### Thin Layer Chromatography

Reference and test solutions of the plant material was prepared following the European Pharmacopoeia 7.4 monograph for *Equisetum* stem (Equiseti herba) test for foreign matter^9^. Due to the limited availability of material from the herbarium specimens in general, only about 20–50 mg of powdered stem was extracted and the amount of methanol used adjusted accordingly. For the test solutions, powdered *Equisetum* stems were extracted with methanol R (VWR BDH Prolabo Chemicals) in the ratio 100 mg/mL. The mixture was heated in a water-bath at 60 °C for 10 min with occasional shaking, allowed to cool and then filtered. The reference solution (a) of *Equisetum palustre* HRS (European Directorate for the Quality of Medicines) was prepared in the same way as the test solutions. Another reference solution (b) was made by dissolving 1.0 mg of caffeic acid R (Sigma), 2.5 mg of hyperoside R (Roth) and 2.5 mg of rutin R (Sigma) in 20 mL of methanol R. For commercial products, 1 g material was extracted with 10 mL methanol R in the same way as the test extracts.

2 μl bands of 8 mm of each solution were applied with a GAMAC nanomat 4 to HPTLC silica gel plates R (5–6 μm; Merck). HPTLC plates were developed over a path of 6 cm using a mobile phase consisting of anhydrous formic acid R (Emsure), glacial acetic acid R (Merck), water R, and ethyl acetate R (Sigma Aldrich) (7.5:7.5:18:67 V/V/V/V). After development, plates were air-dried for 5 min. Detection was achieved by heating at 100 °C for 3 min followed by treatment of the still warm plate with a 10 g/L solution of diphenylboric acid aminoethyl ester R (Roth) in methanol R, and then treatment with a 50 g/L solution of macrogol 400 R in methanol R. Finally plates were air-dried and examined after 10 min in ultraviolet light at 365 nm. System suitability was observed by the appearance of two greenish-blue fluorescent zones from kaempferol glucosides (flavonoids) characteristic of *E. palustre* L. in the reference solution (a) just above the line of application. In the chromatogram of the test solution any greenish fluorescent zones just above the line of application may not be more intense than the corresponding zones (characteristic for *E. palustre*) in chromatogram of the reference solution.

For alkaloid detection, the dried, powdered stem material, which had already been extracted for flavonoid-analysis, was moistened with 10% (1 μL/μg dry plant material). 1 ml dichloromethane (VWR BDH Prolabo) was added, and the mixture was extracted for 24 h at room temperature. 900 μL of the liquid was taken to dryness. The extract was redissolved in 20 μL dichloromethane and applied to a Merck Silica gel 60 F254 TLC plate and eluted in toluene:ethyl acetate:diethylamine (VWR BDH Prolabo; Sigma; Merck) 7:2:1 over 7 cm. 1 mg/mL brucin was used as positive control. The plate was sprayed with 0.15% chloroplatinic acid hydrate (Sigma Aldrich) in a 3% KI solution.

## Additional Information

**How to cite this article**: Saslis-Lagoudakis, C. H. *et al.* Identification of common horsetail (Equisetum arvense L.; Equisetaceae) using Thin Layer Chromatography versus DNA barcoding. *Sci. Rep.* **5**, 11942; doi: 10.1038/srep11942 (2015).

## Supplementary Material

Supplementary Information

Supplementary Dataset 1

## Figures and Tables

**Table 1:** Distinguishing characters between *Equisetum arvense*and*E. palustre*in *matK* and *trnH-psbA* barcodes.

*matK*	*E. arvense* unique substitutions	none
	*E. arvense* substitutions shared with other *Equisetum* species but not with *E. palustre*	With *E. fluvatile*: 265 C, 511 G With *E. diffusum*, *E. fluvatile*: 294 A, 344 C 351 C, 374 C, 397 G, 502 T, 512 C, 567 G, 572 A, 619 T, 636 A, 670 G. With *E. diffusum, E. fluviatile, E. sylvaticum*: 212 T, 462 T. With *E. diffusum, E. fluviatile, E. hyemale, E. sylvaticum*: 372 G.
	*E. palustre* unique substitutions	273 C, 311 A, 391 C, 575 C, 594 C.
	*E. palustre* substitutions shared with other *Equisetum* species but not with *E. arvense*	With *E. laevigatum, E. myriochaetum*: 600 G. With *E. sylvaticum, E. telmateia*: 534 C. With *E. hyemale, E. ramosissimum*: 421 A.
*trnH-psbA*	*E. arvense* unique substitutions	193 A
	*E. arvense* substitutions shared with other *Equisetum* species but not with *E. palustre*	With *E. diffusum, E. fluviatile:* 141 A, 87 A.
	*E. palustre* unique substitutions	124 A, 200 A.
	*E. palustre* substitutions shared with other *Equisetum* species but not with *E. arvense*	With *E. diffusum, E. bogotense, E. sylvaticum*: 121 G, 122 T, 123 A, 125 T, 126 A. With *E. pratense, E. sylvaticum, E. telmateia*: 151 C.

The numbers and substitutions refer to positions in the alignment presented in the Supplementary Information.
