# Supplementary material for: Identification of common horsetail (Equisetum arvense L.; Equisetaceae) using Thin Layer Chromatography versus DNA barcoding
Source: Sci Rep. 2015 Jul 13;5:11942. doi: 10.1038/srep11942 (PMC4499799; doi:10.1038/srep11942)
Supplement: Supplementary Information [file srep11942-s1.doc]

Supplementary Table 1. Details of materials used in the present study. Use in study: * DNA barcoding, + phylogeny of *Equisetum*, # TLC (*E. arvense – E. palustre* comparison), § TLC (all species of *Equisetum*).

| **Study ID** | **Species** | **Collector** | **Collection Number** | **Collection Date** | **Locality** | **Use in study** |
| --- | --- | --- | --- | --- | --- | --- |
| *E. arvense 01* | *Equisetum arvense* L. | M.B. Gerrans | 1345 | 11-07-1967 | Middlesex, UK | * + # |
| *E. arvense 02* | *Equisetum arvense* L. | G. Halliday | 223/82 | 16-08-1982 | Durham, UK | * + # |
| *E. arvense 03* | *Equisetum arvense* L. | C. Madsen | CM002 | 20-10-2013 | Esrum Sø, Denmark | * + # |
| *E. arvense 04* | *Equisetum arvense* L. | C. Madsen | CM001 | 20-10-2013 | Utterslev Mose, Denmark | * + # |
| *E. arvense 05* | *Equisetum arvense* L. | G. Williamson | 106 | 05-07-1974 | Pennsylvania, USA | * + # |
| *E. arvense 06* | *Equisetum arvense* L. | M. Blondeau | TQ92135 | 31-07-1992 | Tasiujag, Canada | * + # § |
| *E. arvense 08* | *Equisetum arvense* L. | S.L. Jury | 12030 | 25-06-1985 | Reading, UK | * # |
| *E. arvense 09* | *Equisetum arvense* L. | B. Deylova | - | 30-07-1976 | Prague, Czech Republic | * # |
| *E. arvense 10* | *Equisetum arvense* L. | M.B. Gerrans | 1345 | 11-07-1957 | Middlesex, UK | * # |
| *E. arvense 11* | *Equisetum arvense* L. | H. Smith | 1197 | 05-09-1921 | China | * # |
| *E. arvense 12* | *Equisetum arvense* L. | V.B. Sochava & V.V. Lipatova | - | 28-07-1956 | Amur District, Russia | * # |
| *E. arvense 13* | *Equisetum arvense* L. | L.A. Gustavsson | 9266 | 10-06-1985 | Phocis, Greece | * # |
| *E. arvense 14* | *Equisetum arvense* L. | K. Hansen | 6030 | 19-08-1960 | Vågø, Faroe Islands | * # |
| *E. arvense 15* | *Equisetum arvense* L. | E. Pobedimova & F. Leisle | 5101 | 14-08-1968 | Leningrad, Russia | * # |
| *E. arvense 16* | *Equisetum arvense* L. | T. I. Alabina | - | 18-09-1944 | Ryazan District, , Russia | * # |
| *E. arvense 17* | *Equisetum arvense* L. | J.Ax. Nannfeldt | 17940 | 13-07-1963 | Gästrikland, Sweden | * # |
| *E. arvense 18* | *Equisetum arvense* L. | H.E. Ahles | 86684 | 02-06-1953 | Massachusetts, USA | * # |
| *E. bogotense 01* | *Equisetum bogotense* Kunth | A. & H. Adsersen | 577 | 25-06-1974 | Fernandina, Galapagos, Ecuador | + § |
| *E. diffussum 02* | *Equisetum diffusum* D. Don | J.F. Rock | 2730 | 02-03-1922 | Southern Yunnan, China | + § |
| *E. fluviatile 01* | *Equisetum fluviatile* L. | G.A. Matthews | - | 21-06-1964 | Hertfordshire, UK | + § |
| *E. fluviatile 02* | *Equisetum fluviatile* L. | O. Skifte & M. Aasen | 50886 | 17-09-1995 | Troms, Norway | + |
| *E. giganteum 01* | *Equisetum giganteum* L. | A. Krapovickas & C.L Cristobal | 17600 | 10-01-1971 | Jujuy, Argentina | + § |
| *E. giganteum 02* | *Equisetum giganteum* L. | G. Hatschbach | 41942 | 18-01-1979 | Curitiba, Brazil | + |
| ***Continued*** |  |  |  |  |  |  |
| *E. hyemale 01* | *Equisetum hyemale* L. | T. Ulvinen & P. Hanhela | - | 23-08-1984 | Pudasjaervi, Finland | + § |
| *E. hyemale 02* | *Equisetum hyemale* L. | T. Barta | 1999-88 | 19-03-1999 | Niederoesterreich, Austria | + |
| *E. laevigatum 02* | *Equisetum laevigatum* A. Braun | A. Nelson | 7693 | 26-07-1900 | Wyoming, USA | + § |
| *E. laevigatum 03* | *Equisetum laevigatum* A. Braun | T. Ranker | EPOB 4520 | 10-06-1993 | Colorado, USA | + |
| *E. myriochaetum 01* | *Equisetum myriochaetum* Schltdl. & Cham. | L. Holm-Nielsen & S. Jeppesen | 280 | 03-06-1968 | Tungurahua, Ecuador | + |
| *E. myriochaetum 02* | *Equisetum myriochaetum* Schltdl. & Cham. | L. Holm-Nielsen & S. Jeppesen | 330 | 04-06-1968 | Tungurahua, Ecuador | + § |
| *E. palustre 01* | *Equisetum palustre* L. | G.A. Matthews | - | 16-06-1961 | North Devon, UK | * + # |
| *E. palustre 02* | *Equisetum palustre* L. | G.A. Matthews | - | 24-05-1973 | Durham, UK | * + # |
| *E. palustre 03* | *Equisetum palustre* L. | P. Paasovaara | - | 06-07-1983 | Kiiminki, Finland | * + # |
| *E. palustre 04* | *Equisetum palustre* L. | O. Seberg | 460 | 15-08-1976 | Gljufrafoss, Iceland | * + # § |
| *E. palustre 05* | *Equisetum palustre* L. | A. Strid et al. | 44398 | 18-07-1997 | Dramas, Greece | * + # |
| *E. palustre 06* | *Equisetum palustre* L. | G. Pettersen | C2944 | 21-05-2013 | Emilia - Romagna, Italy | * + # |
| *E. palustre 08* | *Equisetum palustre* L. | K. Hansen | 1607 | 27-07-1960 | Fjallmannaheyggjur, Faroe Islands | * # |
| *E. palustre 09* | *Equisetum palustre* L. | K. Hansen | 2136 | 03-08-1960 | Strømø, Faroe Islands | * # |
| *E. palustre 10* | *Equisetum palustre* L. | Strid & Mikkelsen | 34887 | 05-05-1993 | Arkadias, Greece | * # |
| *E. palustre 11* | *Equisetum palustre* L. | V.V. Mararov | - | 20-06-1970 | Solnetschno-gorsk, Russia | * # |
| *E. palustre 12* | *Equisetum palustre* L. | P. Monserrat | 3182/71 | 18-06-1971 | Jaca, Spain | * # |
| *E. palustre 13* | *Equisetum palustre* L. | L. Edelberg | 1328 | 13-07-1948 | Nuristan, Afghanistan | * # |
| *E. palustre 14* | *Equisetum palustre* L. | Jon Kaasa | - | 21-08-1971 | Oppland, Norway | * # |
| *E. palustre 15* | *Equisetum palustre* L. | P. Hartvig, C. Baden & S.G. Christiansen | 6081b | 02-07-1976 | Epirus, Greece | * |
| *E. palustre 16* | *Equisetum palustre* L. | L.A. Gustavsson | 9446 | 07-12-1985 | Phocis, Greece | * + # |
| *E. palustre 17* | *Equisetum palustre* L. | N. Maltzev | 7304a | 20-07-1905 | Irkutsk, Russia | * + # |
| *E. palustre 18* | *Equisetum palustre* L. | W.J. Cody | 26663 | 02-07-1980 | Yukon, Canada | # |
| *E. pratense 02* | *Equisetum pratense* Ehrh. | G. Shaughnessy | 78 | 24-06-1992 | Alaska, USA | + § |
| *E. ramosissimum 01* | *Equisetum ramosissimum* Desf. | M.R. Parishani | 14311 | 23-05-2003 | Isfahan, Iran | + |
| *E. ramosissimum 02* | *Equisetum ramosissimum* Desf. | M.G. Gilbert et al. | 7334 | 01-05-1983 | Shewa, Ethiopia | + § |
| ***Continued*** |  |  |  |  |  |  |
| *E. ramosissimum 03* | *Equisetum ramosissimum* Desf. | H.H. Bruun | 43 | 28-02-1990 | Gomera, Canary islands, Spain | + |
| *E. scirpoides 01* | *Equisetum scirpoides* Michx. | M. Blondeau | M-92087 | 12-06-1992 | Quebec, Canada | + § |
| *E. sylvaticum 01* | *Equisetum sylvaticum* L. | K. Nisula & P. Renvall | - | 03-08-1999 | Kuopio, Finland | + § |
| *E. telmateia 01* | *Equisetum telmateia* Ehrh. | E. Bermeier & U. Matthaes | 4113 | 28-07-1994 | Crete, Greece | + |
| *E. telmateia 03* | *Equisetum telmateia* Ehrh. | L. & V. Dalgaard | 13246 | 04-09-1983 | Madeira, Portugal | + § |
| *E. telmateia 04* | *Equisetum telmateia* Ehrh. | J.S. Andersen & A.G. Jensen | 7101 | 19-06-1973 | Sari, Iran | + |
| *E. variegatum 01* | *Equisetum variegatum* Schleich. ex F. Weber & D. Mohr | T. Alm & J. Reiersen | - | 31-08-1990 | Troms, Norway | + |
| *E. variegatum 02* | *Equisetum variegatum* Schleich. ex F. Weber & D. Mohr | P.F. Zika | 1306 | 14-05-1980 | Massachusetts, USA | + |
| *E. variegatum 04* | *Equisetum variegatum* Schleich. ex F. Weber & D. Mohr | T. Neczaeva | - | 15-08-1968 | Sakhalin, Russia | * + § |

Supplementary Table 2. Details of herbal products used in the present study.

| **Study ID** | **Form** | ***E. arvense* mentioned on package** |
| --- | --- | --- |
| A - USA | Tea | Y |
| B - Bulgaria | Tea | Y |
| F - Germany | Tea | N |
| MF - UK | Capsules | Y |
| NA - USA | Capsules | Y |
| NW - USA | Capsules | Y |
| HB - UK | Capsules | N |
| I - UK | Capsules | Y |

Supplementary Table 3. Genbank accession numbers for all material used in the phylogenetic and DNA barcoding analyses.

| **Study ID** | **Genbank accession number** | | | | |
| --- | --- | --- | --- | --- | --- |
|  | *rbcL* | *matK* | *trnH-psbA* | *rsp4* | ITS2 |
| *E. arvense 01* | TBA | TBA | TBA | TBA | - |
| *E. arvense 02* | - | TBA | TBA | TBA | TBA |
| *E. arvense 03* | - | TBA | TBA | TBA | - |
| *E. arvense 04* | TBA | TBA | TBA | TBA | - |
| *E. arvense 05* | - | TBA | TBA | TBA | TBA |
| *E. arvense 06* | TBA | TBA | TBA | TBA | TBA |
| *E. arvense 07* | AY226140 | - | - | AJ583677 | - |
| *E. arvense 08* | - | TBA | TBA | - | - |
| *E. arvense 09* | - | - | TBA | - | - |
| *E. arvense 10* | - | TBA | TBA | - | - |
| *E. arvense 11* | - | TBA | TBA | - | - |
| *E. arvense 12* | - | TBA | TBA | - | - |
| *E. arvense 13* | - | - | TBA | - | - |
| *E. arvense 14* | - | TBA | TBA | - | - |
| *E. arvense 15* | - | TBA | TBA | - | - |
| *E. arvense 16* | - | TBA | - | - | - |
| *E. arvense 17* | - | - | TBA | - | - |
| *E. arvense 18* | - | TBA | TBA | - | - |
| *E. bogotense 01* | TBA | - | TBA | TBA | TBA |
| *E. bogotense 02* | AY226139 | - | - | AJ583678 | - |
| *E. diffussum 01* | AY226141 | - | - | AJ583679 | - |
| *E. diffussum 02* | - | TBA | TBA | - | - |
| *E. fluviatile 01* | TBA | TBA | TBA | TBA | - |
| *E. fluviatile 02* | TBA | TBA | - | TBA | TBA |
| ***Supplementary Table 3 Continued*** |  |  |  |  |  |
| *E. fluviatile 03* | AY226142 | - | - | AJ583680 | - |
| *E. giganteum 01* | - | TBA | - | TBA | - |
| *E. giganteum 02* | TBA | TBA | TBA | - | TBA |
| *E. giganteum 03* | AY226127 | - | - | AJ583681 | - |
| *E. hyemale 01* | TBA | TBA | - | TBA | - |
| *E. hyemale 02* | - | TBA | - | TBA | - |
| *E. hyemale 03* | AY226128 | - | - | AJ583682 | - |
| *E. laevigatum 01* | AY226130 | - | - | AJ583683 | - |
| *E. laevigatum 02* | TBA | TBA | TBA | TBA | TBA |
| *E. laevigatum 03* | - | TBA | TBA | TBA | TBA |
| *E. myriochaetum 01* | TBA | - | - | TBA | TBA |
| *E. myriochaetum 02* | TBA | - | TBA | TBA | TBA |
| *E. myriochaetum 03* | AY226131 | - | - | AJ583684 | - |
| *E. palustre 01* | TBA | - | TBA | TBA | TBA |
| *E. palustre 02* | - | - | TBA | TBA | TBA |
| *E. palustre 03* | TBA | TBA | TBA | TBA | TBA |
| *E. palustre 04* | - | - | TBA | TBA | TBA |
| *E. palustre 05* | TBA | TBA | TBA | TBA | TBA |
| *E. palustre 06* | TBA | TBA | TBA | TBA | - |
| *E. palustre 07* | AY226138 | - | - | AJ583685 | - |
| *E. palustre 08* | - | - | TBA | - | - |
| *E. palustre 09* | - | - | TBA | - | - |
| *E. palustre 10* | - | TBA | TBA | - | - |
| *E. palustre 11* | - | TBA | - | - | - |
| *E. palustre 12* | - | TBA | TBA | - | - |
| *E. palustre 13* | - | TBA | - | - | - |
| *E. palustre 14* | - | TBA | TBA | - | - |
| ***Supplementary Table 3 Continued*** |  |  |  |  |  |
| *E. palustre 15* | - | TBA | TBA | - | - |
| *E. palustre 16* | - | - | TBA | - | - |
| *E. palustre 17* | - | - | TBA | - | - |
| *E. pratense 01* | AY226137 | - | - | AJ583686 | - |
| *E. pratense 02* | TBA | - | TBA | TBA | - |
| *E. ramosissimum 01* | TBA | TBA | TBA | TBA | TBA |
| *E. ramosissimum 02* | TBA | TBA | - | TBA | TBA |
| *E. ramosissimum 03* | TBA | - | - | TBA | TBA |
| *E. ramosissimum 04* | AY226132 | - | - | AJ583687 | - |
| *E. scirpoides 01* | - | TBA | TBA | TBA | - |
| *E. scirpoides 02* | AY226133 | - | - | AJ583688 | - |
| *E. sylvaticum 01* | - | TBA | TBA | TBA | TBA |
| *E. sylvaticum 02* | AY226136 | - | - | AJ583689 | - |
| *E. telmateia 01* | TBA | - | TBA | TBA | - |
| *E. telmateia 02* | AY226135 | - | - | AJ583690 | - |
| *E. telmateia 03* | - | TBA | TBA | TBA | TBA |
| *E. telmateia 04* | - | - | TBA | TBA | TBA |
| *E. variegatum 01* | - | TBA | TBA | TBA | - |
| *E. variegatum 02* | - | TBA | TBA | TBA | - |
| *E. variegatum 03* | AY226134 | - | - | AJ583691 | - |
| *E. variegatum 04* | TBA | TBA | TBA | TBA | - |
| *Herbal product B - Bulgaria* | - | TBA | - | - | - |
| *Herbal product F - Germany* | - | TBA | TBA | - | - |
| *Angiopteris angustifolia* | NC_026300 | - | - | - | - |
| *Ophioglossum reticulatum* | AY138410 | - | - | AF313594 | - |
